# Some Observations on Salpingitis

**Published:** 1917-10

**Authors:** Paul Rockey

**Affiliations:** Portland, Ore.


### SOME OBSERVATIONS ON SALPINGITIS.*

By Paul Rockey, M.D., F.A.C.S., Portland, Ore.

Although these observations include nothing new, they constitute an endorsement of certain views and are offered in the hope that they may be of interest because of the frequency of the condition and the importance of the subiect.

Of the total number of cases of acute salpingitis, one group will make a complete recovery under non-operative treatment, a second group will become chronic, a third will cease to improve at a given subacute point, and a fourth, which is really a very small subdivision of the third, will remain progressively bad acute cases. I have not attempted an accurate estimation of the relative frequency of these groups from our case records but, estimating by general impression, I should say that group four, the progressively bad acute cases, formed much less than 1 per cent. of the whole; that group three, the bad subacute cases, formed less than 5 per cent.; that groups one and two, the acute cases that recover, and the acute cases that go on to chronic types, divide between them at least 95 per cent. As to the relative sizes of these two groups, my impression is that group one is the larger. In recording the belief that more acute salpingitis cases recover than go on to chronic forms, one must remark that the long subsequent histories of some of these cases show that acute salpingitis, which apparently results in recovery, really becomes chronic. And one should consider the chronic cases that make permanent recoveries without operation.

Clinically attacks of salpingitis that are exacerbations of chronic salpingitis are often similar in all respects, both in their phenomena and indications, to initial acute attacks.

The differential diagnosis between acute salpingitis and acute appendicitis is sometimes difficult and occasionally impossible. Where a strong enough doubt remains, I think the patient is entitled to immediate incision. If this proves the appendix is not involved, I think the better judgment is to leave the tubes alone and close the abdomen with or without abdominal or cul de sac drainage, according to the surgeon's judgment in the particular case.

The appendix may be involved in inflammation from without in a case of salpingitis. The right tube or both tubes may be involved in inflammation from without from appendicitis, and subsequently by old adhesions from this source. The differential diagnosis between salpingitis and ectopic pregnancy may be difficult, particularly in the small group of infected ectopic cases.

If a routine practice were made of performing immediate laparotomy on cases of acute salpingitis, the result would be higher mortality and higher morbidity and a loss of many tubes and ovaries that can be saved in whole or part by the methods here advocated, and the performance of many unnecessary operations. Years ago such practice was more common, with results bearing out the statement just made. Evidence is also furnished by the long subsequent histories of many unoperated cases of salpingitis, where not only complete recoveries take place, but pregnancy occurs. It often takes not only better judgment but more nerve to treat these cases expectantly than to subject them to immediate operation. And when the time arises later, if at all, for operation, it requires more operative skill and judgment to save tubes and ovaries than to sacrifice them.

Acute salpingitis cases I would put to bed, elevate the head of the bed about six inches and place an ice bag on the lower abdomen, except in the very small percentage of these cases where it is not well borne. It is safer not to leave the ice bag longer than half an hour in one spot, and generally to discontinue its use during some parts of every twenty-four hours. It is often better to let the patient place it. Why ice bags help I am not sure, but I feel convinced they are of as great or greater value in this class of cases than any other. Give no douches. Give at first only water, subsequently other fluids. Avoid any violent effort to move the bowels.

After temperature has subsided, a hot water bag may be substituted for the ice bag in some cases to aid in more complete resolution, and hot, low pressure saline douches given. A pillow under the knees usualy gives comfort. The patient should, of course, remain in bed and, while the care of such a case may be carried out satisfactorily in her home, it is generally better done in the hospital. Let me emphasize the fact that the success of non-operative as well as every other kind of treatment depends on attention to detail.

An incomplete, infected abortion is entitled to have the uterus properly emptied. An abscess in the posterior cul de sac is entitled to drainage. The operation of cul de sac drainage of a pelvic abscess should be undertaken only where the surgeon is prepared to proceed with immediate laparotomy, if it should be indicated.

Some of the cases of pelvic infection sometimes following child birth and miscarriage but more often criminal abortion, are among the most severe cases to which the surgeon is called. Often the infection soon spreads to involve all of the tissues of the pelvis and in addition becomes a general bacteremia. In these cases retained secundines should be removed from the uterus and gross abscesses, if any occur, drained thru the posterior cul de sac or thru the abdominal wall, but otherwise non-operative offers these unfortunate women better chances than operative treatment.

The surgeon's judgment decides when he is dealing with a case that improves only to a certain subacute point. Then these cases are operated on best thru a medium incision, probably with the removal of both tubes. But even here is it often possible to leave at least a little open stump of one tube, so that the woman may have some chance of child-bearing, even if it be a much smaller chance than the average. Probably at least one ovary can be saved. Drainage may or may not be indicated, abdominal or cul de sac, generally the latter. Close the deep fascia with interrupted chromic sutures, the skin and subcutaneous tissue with interrupted silk worm suture. A small twisted cat gut or gutta percha drain may be placed at the lower angle of such a wound to drain the subcutaneous space.

In this smaal group of subacute cases requiring operation, it will be necessary to separate adhesions. These become firmer the longer the case waits and therefore present greater operative difficulty. This is one factor to be taken into account in deciding when to operate. The very rare acute operative cases of group four should always be drained, most likely with gauze and gutta percha abdominal drain.

I wish to say a word about cul de sac drainage in cases with abdominal incision. The method of freeing adhesions in the cul de sac so there is plenty of room, packing a single piece of gauze of sufficient length, closing the abdomen, then doing a posterior coloptomy and pulling down the gauze is most satisfactory when properly performed. This method is not only of value in subacute cases where pus in encountered, but also in subacute and chronic cases to take care of the oozing that may follow the separation of extensive adhesions. I will not go into the subject of the detailed post-operative care of these cases.

Chronic salpingitis cases may be operated on because of sterility, where due investigation points out the tubes as the probably cause. Such celiotomies may be done thru vertical, transvers vertical or Pfannenstial incisions. They may consist in removing part of one or both tubes. But in any case leave the ends of the tubes open and in such a manner that they may remain open. Where both tubes are stenosed at the cornua, it has been proposed to resect this stenosed portion and transplant the patent portion, distal to this, into the cornu. In these cases a hard nodule is often observed in the tube just outside the cornu.

It is probable that some of the cases of one-child sterility are cases where salpingitis followed post-partum infection, and some of these cases that conceive again after a lapse of years are probably the ones where the inflammatory process disappeared, and the patency of a tube became re-established.

It may be necessary to free dense adhesions in some chronic salpingitis operations. It may be advisable to do a suspension. Formerly we used the Gilliam suspension or a modification of it and had good results but we now do Baldy-Webster suspensions as a rule. In women past the child-bearing age ventral fixations are occasionally indicated rather than suspensions. Chronic cases may be operated on because of repeated exacerbations. They are better operated on in periods of remission.

Operation may be indicated by more or less continued pain, due to pelvic adhesions without inflammatory exacerbation. Some of these cases are adherent retroversion cases. Retroversion of the uterus, per se, is not an indication for operation. This is true even where various complaints are traceable to it but where non-operative measures fail to correct the retroversion or complaints operation may be indicated.

Repeated examinations and careful observation are important in passing judgment on cases of chronic salpingitis, with careful attention to the details of non-operative treatment, directed not only toward the pelvis but also toward the general health and hygiene. Meanwhile the surgeon should not only try to picture exactly in his mind the pathology of the case but also the logical relation between the pathology and the symptoms. As time goes on we shall be able to explain many symptoms more exactly. For instance, we may expect to find out why some salpingitis cases have pain referred under the arcehs of the ribs, especially the right.

There are undoubtedly cases of chronic salpingitis that will continue to cause discomfort until buth tubes are completely removed. The experienced surgeon may in his judgment select these cases for complete removal at a first laparotomy, if the patient is at the end of the child-bearing period but, where this prognosis is in doubt as it usually is and, where the patient is younger, it seems better to leave at least a part of one tube, knowing that this might require removal subsequently. Some cases of chronic salpingitis will continue to give trouble until the intramural portion of the tube at the cornu is also excised. Salpingitis of all types occasionally occurs in rather young girls and in old women.

It is true that in late subacute cases the pus in pyosalpinx and tubo-ovarian abscess is often sterile and more often practically innocuous, if not sterile, but occasionally it is virulent. I think this is more apt to be so in cases following postpartum or abortion infection, where the infecting organism is not the gonococcuc but generrally the streptoccoccus. Therefore, at operation all pelvic pus should always be dealt with carefully, because it is sometimes virulent.

Where there is a chance of encountering gross pus in a salpingitis operation, it seems safer to begin with the patient in the horizontal position and continue in that position until the danger is past of pus running up, as it might in the Trendelenburg position. It is to be remembered that, with a little more mechanical difficulty, salpingitis operations can be done entirely with the patient in the horizontal position.

Whatever the infecting organism in salpingitis, mixed infections are common. Some cases of diffuse or general tuberculous peritonitis appear to have had their origin in tuberculous salpingitis. On the other hand, it is probable that the tubes are sometimes involved in cases of tuberculous peritonitis, originating elsewhere. Various types and stages of tuberculous salpingitis are observed, varying from a few small tubercles to massive caseation. The judgment of what to do with tuberculous tubes in a given operation must depend on all the factors of the case.

Every effort should, of course, be made to save ovarian tissue. It is usually safe and feasible, even in subacute cases, to separate adhesions between the tube and the ovary, about which it is often coiled.

Under the head of prevention of salpingitis should be mentioned not only the prevention of postpartum infections but also the prevention and proper treatment of infections after miscarriages and criminal abortions and the treatment of gonorrhea. I wonder how often the common gonorrheal and other vulvovoginitis of infancy and childhood is responsible for endometritis and salpingitis of later years. It seems to me that hysterectomy for chronic endometritis must be very rarely indicated.

In conclusion, group one, the acute cases that recover if given the benefit of non-operative treatment, require no operation at all. Group two, the acute cases that become chronic, may require suitable operation at some future time. But if this time comes, the operation will save life and health, and the function of ovaries and tubes that would likely have been sacrificed in an acute operation. Group three, the small class of subacute cases that do not improve, require operation. Group four comprises the very rare cases of acute salpingitis that do not improve.

The surgeon's duty is not made up merely of performing operation, but of careful diagnosis, sound judgment, close observation and scrupulous attention to the details of non-operative as well as anteoperative, operative and postoperative treatment, in the light of the general rule that he has first to save life and second to save health and function. It is our observation in this view of surgery, that the foregoing are proper principles for the treatmnt of salpingitis.—Northwest Medicine.

